# Comparative analysis of the value of diffusion kurtosis imaging and diffusion-weighted imaging in evaluating the histological features of endometrial cancer

**DOI:** 10.1186/s40644-019-0196-6

**Published:** 2019-02-14

**Authors:** Wei Yue, Nan Meng, Jing Wang, Wenling Liu, Xuejia Wang, Minghuan Yan, Dongming Han, Jingliang Cheng

**Affiliations:** 1 Department of MRI, The First Affiliated Hospital, Zhengzhou University, 1 Janshe East Road, Zhengzhou, 450000 People’s Republic of China; 2 Department of MRI, The First Affiliated Hospital, Xinxiang Medical University, 88 Jiankang Road, Weihui, 453100 People’s Republic of China

**Keywords:** Diffusion kurtosis imaging, Diffusion-weighted imaging, Endometrial cancer

## Abstract

**Purpose:**

This study evaluated and compared the performances of diffusion kurtosis imaging (DKI) and diffusion-weighted imaging (DWI) for diagnosing and histologically grading endometrial cancer.

**Materials and methods:**

In this retrospective study, DKI and DWI data for 61 patients with endometrial cancer and 30 patients with a normal endometrium were analyzed, and the mean kurtosis (MK), mean diffusion coefficient (MD) and apparent diffusion coefficient (ADC) values for the endometrial cancer tissue and normal endometrial tissue were acquired. The parameters for the normal endometrium group (G0) and the endometrial cancer groups (G1, G2 and G3) were compared and analyzed. The receiver operating characteristic (ROC) curve was used to evaluate each parameter’s diagnostic accuracy and threshold. Spearman correlation analysis was used to analyze the correlations between all parameters and histological grades.

**Results:**

The MK values for the G0, G1, G2 and G3 groups increased gradually, while the MD and ADC values decreased gradually. Except for the differences in the ADC values between G0 and G1, the differences among the groups were statistically significant (*P* < 0.05). The MK values had the highest diagnostic accuracy in differentiating G0 and (G1 + G2 + G3), G0 and G1, G1 and G2, and G2 and G3 (AUC = 0.93, 0.76, 0.91, 0.91, P < 0.05). MK was maximally correlated with histological grade, followed by MD and ADC (MK > MD > ADC; r = − 0.85, + 0.82, + 0.76, *P* < 0.01).

**Conclusion:**

Both DKI and DWI can be used to evaluate the diagnosis and histological grading of endometrial cancer. Compared with DWI, the DKI model is a more complete mathematical model with more sensitive parameters, which can more effectively evaluate the pathological and physiological characteristics of endometrial cancer.

## Background

Endometrial cancer (EC) is one of the most common malignancies among women, with a high incidence and mortality. It is a life-threatening disease among women, with its age of onset becoming younger [1, 2]. Research has shown that the histological grade for EC is closely associated with planning the treatment and determining the prognosis [3]. Among patients with International Federation of Gynecology and Obstetrics (FIGO) stage I EC, only 10% of patients with low-grade endometrioid carcinoma (grade 1 or grade 2) are expected to have nodal metastasis, but close to 18% of patients with high-grade endometrioid carcinoma (grade 3) are expected to have nodal metastasis [4]. Traditional multiple punch biopsy under vaginoscope is easily affected by factors such as lesion size, sampling accuracy, and operator experience, with certain differences between the results and final pathology. Studies have shown that 19% of patients diagnosed with grade 1 endometrial adenocarcinoma by multiple punch biopsy under vaginoscope were upgraded after total hysterectomy [5]. Hence, accurately evaluating the pathology type and grade of differentiation of EC is beneficial for patient prognosis.

Routine diffusion-weighted imaging (DWI) is based on the Gaussian distribution of the diffusion motion of water molecules and can measure the mobility of water molecules diffusing throughout tissues [6]. Some studies have shown that ADC values are valuable for diagnosing tumor and assessing the degree of differentiation [7, 8] because there is a reduction in extracellular space as tumor cells pack more tightly, which lowers ADC values. However, Kishimoto K et al. [9] and Bharwani N et al. [10] found that DWI is difficult to distinguish between different differentiated endometrial cancers. With the development of DWI, a series of more advanced DWI technologies, such as structural anisotropy (diffusion tensor imaging [DTI]), microvascularity (intravoxel incoherent motion [IVIM]) and microstructural complexity (diffusion kurtosis imaging [DKI]), have emerged in recent years, providing more disease information and facilitating disease diagnosis [11–14]. The IVIM imaging is a technique with the potential for simultaneously assessing both tissue perfusion and diffusion by using a single diffusion weighted imaging with a different number of b values. DTI extends routine diffusion imaging module to characterize the orientational variability of the diffusion process, allowing assessment of diffusion directionality or anisotropy [6]. And DKI has a unique advantage in reflecting the true diffusion movement of water molecules. Many studies have shown that due to diffusion barriers, such as cell membranes, organelles, and intercellular space restrictions, water diffusion in vivo is more sophisticated than a standard Gaussian distribution [15]. Additionally, DKI is based on a non-Gaussian diffusion model that better addresses restricted water diffusion within the complex microstructure of biological tissues. Theoretically, DKI can quantify the diffusion state of non-Gaussian water molecules in tissues, thus correcting the offset of the Gaussian model [16, 17] and improving the detection of lesions.

Currently, studies on the pathological grading of EC have focused on a single technique. For example, Toba et al. [18] found that FA values in DTI can show myometrial invasion of EC; Fasmer et al. [19] found that dynamic contrast-enhanced MRI (DCE-MR) helps to assess the pathological type of EC, etc. Meanwhile, comparative studies on DWI and DKI mainly focus on the diagnosis and differential diagnosis of head and neck cancer [20] and prostate cancer [8, 21]. There are few comparative studies of DWI and DKI in EC [22]. The purpose of the present study was to evaluate the feasibility of DKI in diagnosing and histologically grading EC and to determine whether DKI is superior to DWI.

## Methods

### Subjects

The research was approved by the Ethics Committee of our hospital, and all patients signed informed consent forms before scanning. Patients with primary EC who underwent MRI examinations at our hospital from July 2017 to August 2018 were enrolled in this study. Inclusion criteria were 1) menopausal patients and 2) surgery was performed within 1 week after the MRI scan, with confirmed pathology results. Exclusion criteria were 1) patients who received radiotherapy or chemotherapy before the MRI scan, 2) patients whose experimental sequence was incomplete or showed significant motion or metallic artifacts, and 3) patients who had rare uterine tumors such as serous adenocarcinoma, carcinosarcoma, or mixed adenoneuroendocrine carcinoma. Inclusion criteria for the control group were 1) menopausal patients, 2) no relevant clinical symptoms, such as abnormal uterine bleeding, with MRI images showing normal endometrium, and 3) images being clear and suiting the needs of diagnosing and image postprocessing. The patients with normal endometrium were used as the control group (G0), and the pathology results for the control group were used as the gold standard. Patients with EC were classified into three groups: the G1 group (less than 5% of the ovarian epithelium showed mild dysplasia), G2 group (6–50% of the ovarian epithelium showed mild dysplasia) and G3 group (more than 50% of the ovarian epithelium showed severe dysplasia). If the abovementioned G1 and G2 groups had severe dysplasia, then their tumor grades were adjusted a grade higher.

### Image acquisition

A pelvic magnetic resonance (MR) scan was performed using a 3.0 T MR scanner (Discovery MR750, GE Healthcare, Milwaukee, Wisconsin), and a 32-channel phased-array torso coil was used for imaging the pelvis. The scanning range was from the anterior superior iliac spine to the symphysis pubis. Before the examination, patients were required to have a full bladder, and a gel sponge for vaginal packing was applied to ensure that the uterus was in a moderately forward position for observation and scanning of the lesion. MR imaging protocols were created with the following sequences: sagittal T2-weighted imaging (T2WI) without fat suppression and coronal fat-suppressed T2WI. These protocols used the following parameters: field of view (FOV): 36 × 28 cm^2^; slice thickness: 6.0 mm; spacing: 2.0 mm; and number of slices: 20. Axial fat-suppressed T2WI and T1-weighted imaging (T1WI) without fat suppression were performed. These protocols used the following parameters: FOV: 36 × 28 cm^2^; slice thickness: 5.0 mm; spacing: 1.0 mm; and number of slices: 20. Axial oblique DWI was performed with the following parameters—FOV: 36 × 28 cm^2^, slice thickness: 5.0 mm, spacing: 1 mm, b-value: 800 s/mm^2^, NEX: 4. Spin-echo echo-planar imaging (SE-EPI) was used to acquire the axial oblique DKI sequence with the following parameters: FOV: 36 × 28 cm^2^; slice thickness: 5 mm; spacing: 1 mm; TR: 2500 ms; TE: 79.3 ms; matrix: 128 × 128; NEX: 2; b-values: 0, 500, 1000, 1500, and 2000 s/mm^2^; and 30 uniformly distributed dispersion directions.

### Image processing and analysis

#### Image processing

All images obtained by DWI and DKI were transferred to a workstation (Advantage workstation 4.6, GE Healthcare, Milwaukee, Wisconsin) and postprocessed using the DKI and DWI processing toolboxes available within the Functool software.

For the DKI model, the relationship between the signal intensity of DKI and b factors can be expressed by the eq. S(b) = S0. exp . (−b · Dapp + b2Dapp2Kapp/6). For the DWI model, the relationship between the signal intensity of DWI and b factors can be expressed by the eq. S(b) = S0. exp(−b · ADC).

S is the signal intensity (arbitrary unit), and b is the b-value (s/mm^2^).

We applied the signal intensity data of five b-values (b = 0, 500, 1000, 1500, and 2000 s/mm^2^) to obtain the DKI parameters (MD, MK). MD is the corrected apparent diffusion parameter of the Gaussian distribution (10^− 3^ mm^2^/s), and MK is the apparent kurtosis coefficient (dimensionless). We applied the signal intensity data of two b-values (b = 0, 800 s/mm^2^) to obtain the DWI parameters (ADC). ADC is the apparent diffusion coefficient of the Gaussian distribution (10^− 3^ mm^2^/s) using the conventional mono-exponential model. Therefore, three independent parametric maps were obtained for each patient.

#### Measurement of parameters

One attending physician and one associate chief physician (with 10 and 15 years of experience in diagnosis of gynecological disease images, respectively) who were blinded to the clinical and pathological data independently read and measured the apparent diffusion coefficient (ADC), mean diffusion coefficient (MD), and mean kurtosis (MK) values.

#### The region of interest (ROI) placement

The ROI was manually selected layer by layer on the level of the DWI/DKI sequence containing the tumor tissue. The average values for each ROI parameter were calculated as their final values. Reference standards were as follows: 1) the ROI should contain solid tumor issues as much as possible; 2) ROI placement was chosen to avoid necrotic, cystic, or bleeding regions. In the normal control group, the ROI was also selected layer by layer, and its drawing and calculation were the same as above.

### Statistical analysis

All statistics were performed using SPSS 23.0 (SPSS, Chicago, IL, USA) and MedCalc version 11.1.1.0 for Windows (MedCalc software, Mariakerke, Belgium). Bland-Altman plots were used to check and evaluate the consistency of the results measured by the 2 physicians. The Kolmogorov-Smirnov test was used to check and evaluate the normal distribution of the measurement data and normally distributed data were expressed as $\overline{\mathrm{x}}\pm \mathrm{s}$. Analysis of variance (ANOVA) was used to compare the MD, MK and ADC values for G0, G1, G2, and G3. The individual sample t test was applied for between-group analyses. The correlation between each parameter and histological grade was analyzed by Spearman’s correlation coefficient. The receiver operating characteristic (ROC) curve was generated to evaluate each parameter’s diagnostic accuracy and threshold. Differences among the data were considered statistically significant at *P* < 0.05.

## Results

In total, 91 patients were selected in this research, including 61 patients with EC, with a mean age of 60.53 ± 8.99 years (17 in G1, 63.41 ± 9.84 years; 24 in G2, 59.58 ± 9.85 years; and 20 in G3, 57.85 ± 6.3 years), and 30 patients with normal endometrium, with a mean age of 57.40 ± 9.58 years. There were no statistically significant differences in age between the groups (Tables 1 and 2).

**Table 1:** Parameter comparison between the endometrial cancer group and the normal endometrium group

Groups	Sample Size(case)	Age(year)	MK	MD(×10^−3^ mm^2^/s)	ADC(× 10^− 3^ mm^2^/s)
Endometrial cancer group	61	60.08 ± 8.97	0.82 ± 0.09	1.14 ± 0.11	1.01 ± 0.19
Normal endometrium group	30	57.40 ± 9.58	0.67 ± 0.04	1.39 ± 0.19	1.28 ± 0.19
t value		−1.28	− 11.19	7.04	6.49
*P* value		0.21	P < 0.01	P < 0.01	P < 0.01

**Table 2:** Comparison of parameters between groups G1, G2 and G3

Groups	Sample Size(case)	Age(year)	MK	MD(×10^−3^ mm^2^/s)	ADC(× 10^− 3^ mm^2^/s)
G1	17	63.41 ± 9.84	0.72 ± 0.05	1.26 ± 0.06	1.19 ± 0.14
G2	24	59.58 ± 9.85	0.82 ± 0.05	1.14 ± 0.07	1.01 ± 0.13
G3	20	57.85 ± 6.30	0.91 ± 0.04	1.03 ± 0.05	0.83 ± 0.11
F value		1.88	23.52	46.16	8.93
P value		0.16	P < 0.01	P < 0.01	P < 0.01

**Fig. 1:**
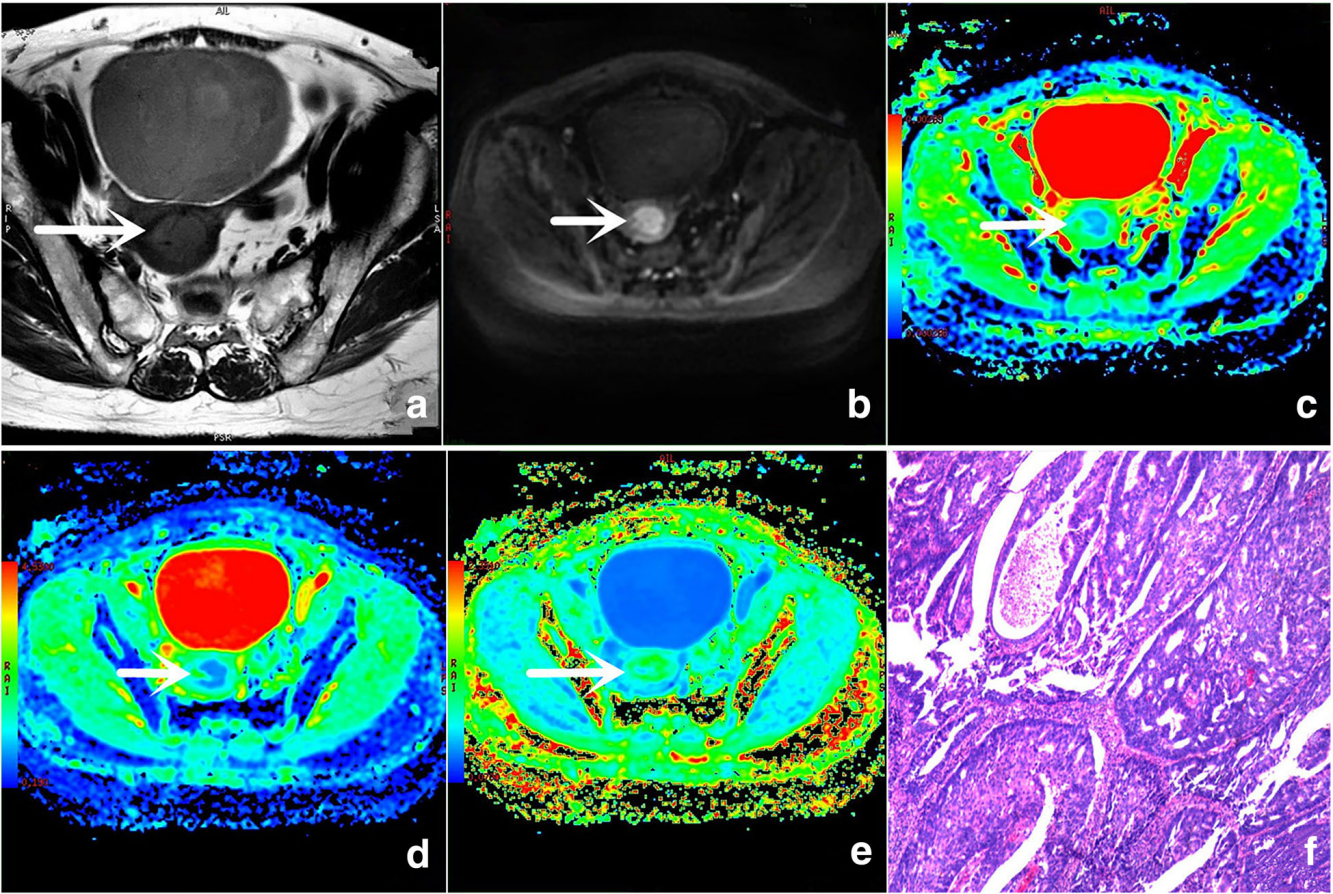
A 54-year-old woman with grade 2 (G2) endometrial cancer. **a** Small FOV, axial oblique T2WI shows a slightly higher signal intensity in the lesion (arrow). **b** Conventional FOV, axial oblique DWI (b value: 600 s/mm^2^) shows a high signal intensity in the lesion (arrow). **c** False color map of the ADC shows a slightly lower light blue signal in the lesion (arrow). **d** False color map of the DKI-derived MD shows a light blue signal in the lesion (arrow). **e** False color map of the MK shows a slightly higher green signal in the lesion (arrow). **f** Histological tissue slice (hematoxylin and eosin [H/E] × 400) shows that the tumor cells are in a gland-like arrangement with mild abnormalities.

The original images generated by the DKI and DWI sequences and each parameter’s false color images are shown in Fig. 1.

**Fig. 2:**
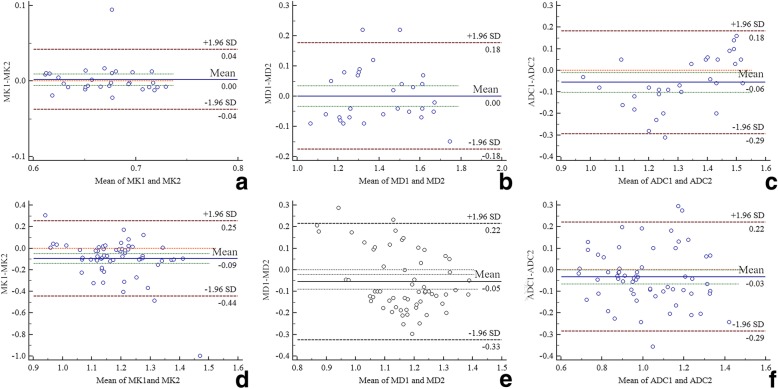
Bland-Altman diagram of MK/MD/ADC values measured by 2 observers (**a b** and **c** belong to the normal endometrium group; **e f** and **g** belong to the EC group, 1 represents the first observer and 2 represents the second observer)

Bland-Altman analysis showed that the MK, MD, and ADC values measured by both observers were in agreement: normal group, Fig. 1a MK 29/30 (96.7%), Fig. 1b MD 28/30 (93.3%) and Fig. 1c ADC 29/30 (96.7%); EC group, Fig. 1d MK 59/61(96.7%), Fig. 1e MD 59/61 (96.7%), and Fig. 1f ADC 58/91 (95.1%) (Fig. 2). Hence, the data acquired by the deputy chief physician were used as the input.

**Fig. 3:**
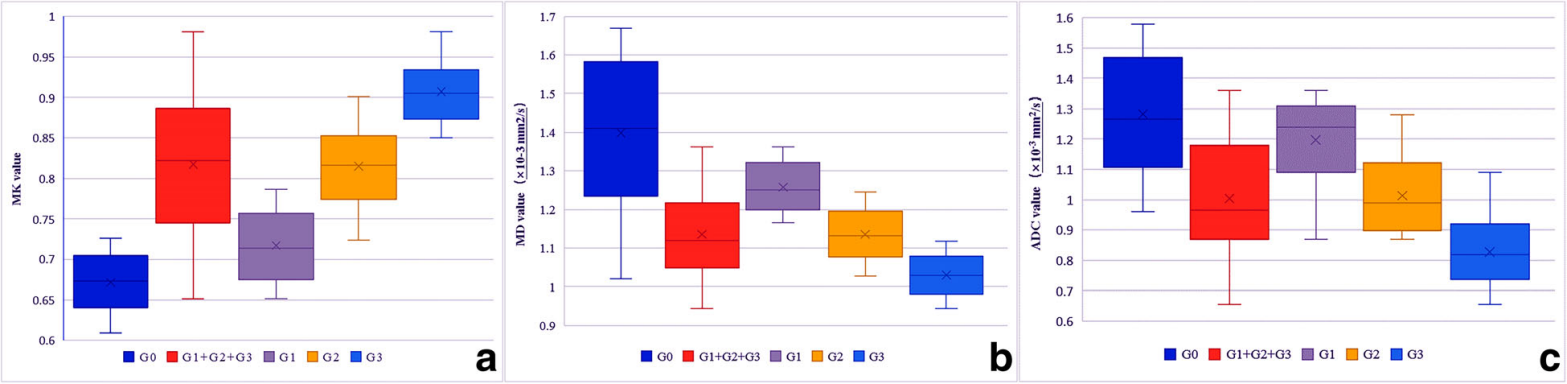
Box chart of MK/MD/ADC values in different groups (dark blue color represents G0, red color represents G1+G2+G3, purple color represents G1, yellow color represents G2 and light blue color for G3)

The MK value was higher in the (G1 + G2 + G3) group than that in the G0 group (*P*<0.01) (Table 1, Fig. 3a), and the MD and ADC values were lower in the (G1 + G2 + G3) group than that in the G0 group (*P*<0.01) (Table 1, Fig. 3b, c). Intergroup comparison showed that the MK value was highest in group G3, followed groups G2 and G1 (G3 > G2 > G1) (Table 2, Fig. 3a), whereas the MD and ADC values were highest in G0, followed by groups G1, G2, and G3 (G3 < G2 < G1) (Table 2, Fig. 3b, c). The difference in ADC values between groups G0 and G1 were not significant (*P* = 0.09) (Table 2, Fig. 3c). The differences in the MK, MD and ADC values among all tumor grade groups were significant (Table 2, Fig. 3a, b, c).

**Fig. 4:**
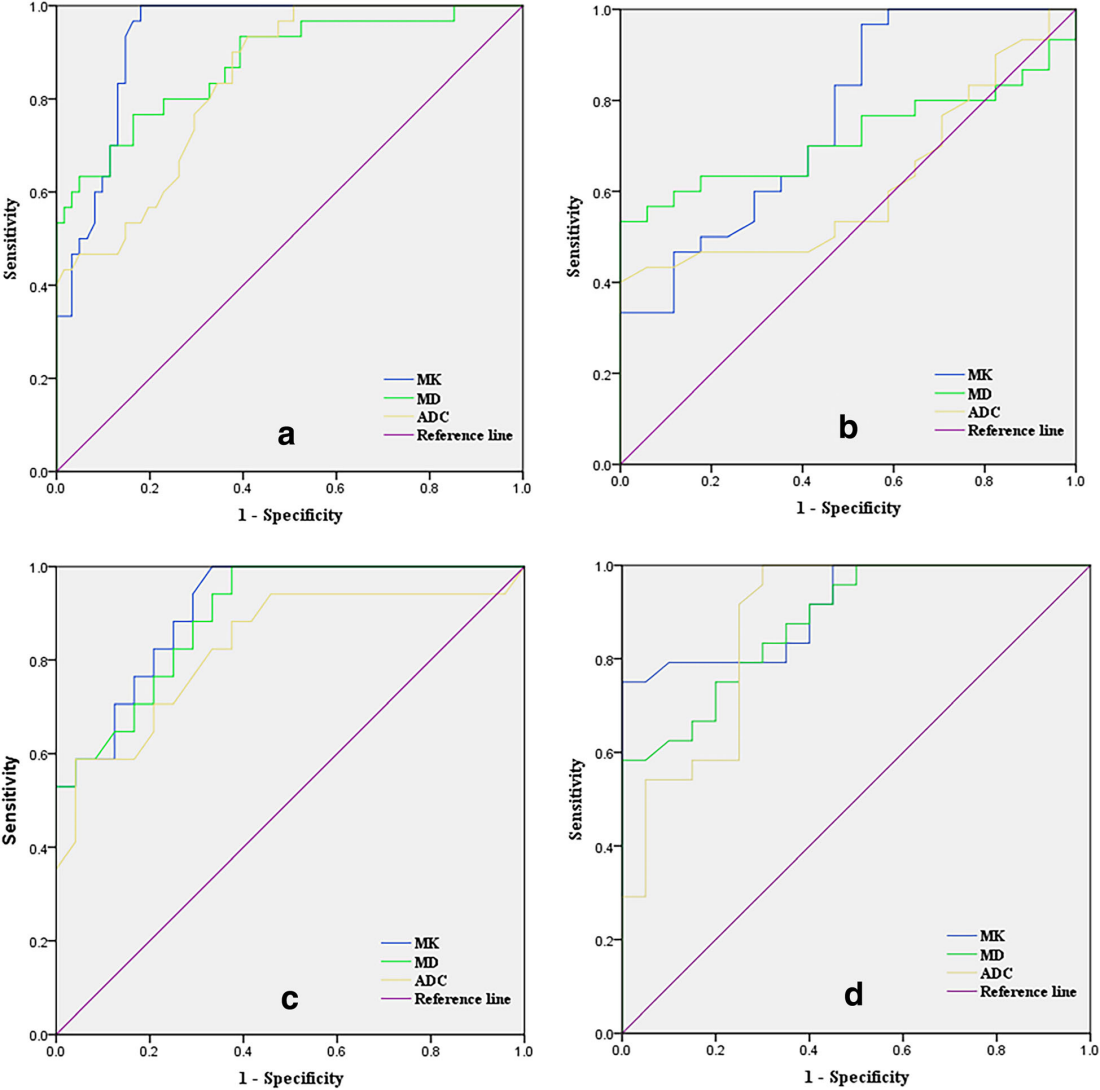
**a** ROC curve for all parameters in the normal endometrium group (G0) and the endometrial cancer groups (G1, G2, G3); **b** ROC curve for parameters in groups G0 and G1; **c** ROC curve for parameters in groups G1 and G2; **d** ROC curve for parameters in groups G2 and G3

The ROC curves for the MK, MD, and ADC values for identifying EC and different grade groups are shown in Fig. 4. The AUC value of MK was the highest, followed by those of MD and ADC (MK > MD > ADC; Tables 3, 4, 5, and 6).

**Table 3:** Comparison of ROC curves for the normal endometrium group and the endometrial cancer groups

Parameters	AUC(95%CI)	*P* value	Threshold	Sensitivity (%)	Specificity (%)	Youden’s Index (%)
MK	0.93(0.88–0.98)	< 0.01	0.73	96.7	82.0	78.7
MD	0.88(0.79–0.96)	< 0.01	1.24	76.7	83.6	60.3
ADC	0.84(0.76–0.92)	< 0.01	1.06	90.0	62.3	52.3

**Table 4:** Comparison of ROC curves for groups G0 and G1

Parameters	AUC(95%CI)	*P* value	Threshold	Sensitivity (%)	Specificity (%)	Youden’s Index (%)
MK	0.76(0.61–0.90)	0.004	0.72	96.7	47.1	43.8
MD	0.72(0.57–0.86)	0.014	1.35	56.7	94.1	50.8
ADC	0.62(0.46–0.78)	0.188	/	/	/	/

**Table 5:** Comparison of ROC curves for groups G1 and G2

Parameters	AUC(95%CI)	*P* value	Threshold	Sensitivity (%)	Specificity (%)	Youden’s Index (%)
MK	0.91(0.83–0.99)	< 0.01	0.79	94.1	71.8	65.9
MD	0.89(0.80–0.99)	< 0.01	1.17	94.1	66.7	60.8
ADC	0.83(0.69–0.97)	< 0.01	1.14	58.8	95.8	54.6

**Table 6:** Comparison of ROC curves for groups G2 and G3

Parameters	AUC(95%CI)	*P* value	Threshold	Sensitivity (%)	Specificity (%)	Youden’s Index (%)
MK	0.91(0.83–0.99)	< 0.01	0.85	75.0	95.0	70.0
MD	0.88(0.79–0.98)	< 0.01	1.11	58.3	95.0	53.3
ADC	0.87(0.77–0.98)	< 0.01	0.87	95.8	70.0	65.8

The correlation between parameters and histological grade was maximal for MK, followed by MD and ADC (MK > MD > ADC; r = − 0.85, + 0.82, + 0.76, *P* < 0.01).

## Discussion

### Evaluation of DKI and DWI in the diagnosis and histological grading of endometrial cancer

The results of this study show that the MK, MD and ADC values can be used to diagnose EC and evaluate its pathological grade. The MK, MD and ADC values essentially reflect water diffusion behavior in tissues, the MK value represents the degree of water molecule diffusion motion that deviates from the Gaussian distribution, and the MD and ADC values reflect the degree of a hindrance to water diffusion. The greater the water diffusion hinderance, the more the water diffusion deviates from Gaussian distribution. As the MK value increases, the MD and ADC values decrease by increasing the hindrance. Many factors affect water diffusion in biological tissues, including cell density, the nucleocytoplasmic ratio, and the ratio between free and bound water [23, 24]. These factors are normally stable, and the above parameters are approximately constant. However, abnormal cancer cell proliferation causes various factors to become imbalanced and further hinders normal water molecule movement. This imbalance and hindrance increase with the increasing degree of malignancy of malignant tumors. Gradually, the parameter values will differ between the normal and cancer tissues and between the histological grades of the cancer tissues. The results from our studies are consistent with those of previous studies [25, 26]. In addition to comparing groups G0 and G1 + G2 + G3, groups G1, G2, and G3 were also individually compared with G0. It was difficult to discriminate the G0 and G1 groups using the ADC value, but the MK and MD values could be used to differentiate these groups, indicating that DKI can better diagnose cancer. Our results also suggested that the diagnostic capabilities of DWI and DKI might have been overestimated if only groups G0 and (G1 + G2 + G3) were compared because groups G2 and G3 had more cases.

### Diagnostic performances of DWI and DKI

In diagnosing and histologically grading EC, our study showed that MK values had the maximum AUC and the most significant correlations with the AUC; MD was second, followed by ADC. The respective comparisons between G1, G2, and G3 and G0 showed that the MK and MD values effectively discriminated G0 from G1, while the ADC values could not. Hence, DKI can diagnose EC and differentiate different histological tumor grades more effectively than DWI. This result is consistent with the conclusions of Bai [27] and Yu et al. [28] on glioma and rectal cancer, respectively.

Both DWI and DKI reflect the diffusion behavior of water molecules; however, they differ. The mathematical model and parameter algorithm for DWI are calculated based on the Gaussian distribution of water diffusion, which does not conform to the true diffusion of water molecules in biological tissue. The lesions displayed by DWI are closely associated with the b-value [29, 30]. When b is less than 1000 s/mm^2^, water movement in the tissue seems to conform to the Gaussian distribution, since some minor factors that hamper water movement is difficult to detect, such as cell membranes and organelles. When b is no less than 1000 s/mm^2^, the effects of these minor factors become apparent, and water movement gradually deviates from the Gaussian distribution. Therefore, due to its own limitations, DWI can only roughly evaluate the lesion. Ignjatovic et al.’s study on glioma cancer [31] demonstrated that cancer diagnosis using DWI was unsatisfactory. Although DWI has some ability to diagnose cancer and assess tumor grades, it cannot discriminate normal brain tissue from low-grade glioma cancer tissue and cannot differentiate two adjacent glioma tumor grades. As mentioned previously, imaging with higher b-values can better detect the true status of water movement. DKI is such an imaging method for detecting water movement with high b-values. Compared with DWI, DKI has a higher b-value (up to 2000 s/mm^2^ in our study) and provides a more complete model for data fitting [32, 33]. Therefore, DKI can sensitively detect minor factors that affect water movements, such as cell membranes and organelles. In addition, the comparison between two DKI-derived parameters, MK and MD, showed that MK is always superior to MD, possibly because MK is a fourth-order three-dimensional tensor parameter and can describe water movement in a more advanced way than MD [34].

### Limitations

Our study had some limitations. 1) Studies were only focused on EC with different histological grades, but other rare subtypes were not included, such as carcinosarcoma; therefore, the representativeness of the results is low. In the future, more pathologies will be included for a more detailed study. 2) The DKI scanning parameters were suboptimal, as there are few studies on the application of DKI for diagnosing EC. Further estimation of the diagnostic performance of the DKI model and conventional ADC model by using DKI-optimal and ADC-optimal protocols is needed in future prospective investigations. 3) ROI placement was chosen manually to avoid the cystic and necrotic areas, thus artificially reducing the tumor tissue’s heterogeneity and possibly affecting the diagnostic accuracy of MK. In our next study, we will consider using histogram analysis to improve the accuracy.

## Conclusion

In summary, DKI technology is based on a non-Gaussian model and has shown advantages in diagnosing and histologically grading EC compared with conventional DWI. This indicates that DKI can be applied to noninvasively assess cancer pathology. Therefore, DKI can be an important supplement to conventional MRI, providing more diverse and detailed information for diagnosing EC.

## Abbreviations

ADC: Apparent diffusion coefficient
AUC: Area under curve
DKI: Diffusion kurtosis imaging
DWI: Diffusion weighted imaging,
EC: Endometrial cancer
FOV: Field of view
MD: Mean diffusion coefficient
MK: Mean kurtosis
MRI: Magnetic resonance imaging
ROC: Receiver operating characteristic
ROI: Region of interest
